# An audit to assess the measurement of Body Mass Index (BMI) and referral to the dietetics service following admission to the general adult inpatient wards in Mersey Care NHS Foundation Trust

**DOI:** 10.1192/bjo.2021.258

**Published:** 2021-06-18

**Authors:** Declan Hyland, Millie Prime, Rabia Khaliq

**Affiliations:** 1 Consultant Psychiatrist, Clock View Hospital, Liverpool, Mersey Care NHS Foundation Trust; 2 5th year medical undergraduate, University of Liverpool

## Abstract

**Aims:**

This audit aims to establish whether patients have their BMI measured and recorded following admission to the general adult inpatient wards in Mersey Care NHS Foundation Trust and whether, in those with a BMI >30 kg/m2, or >28 kg/m2 in those with weight-related comorbidities, they are referred to the dietetics service for input.

**Background:**

Obesity has an increased prevalence in those with mental disorder. There are many factors that influence this, e.g. sedentary lifestyle and poor dietary intake. Medication prescribed to treat mental disorders may increase risk of weight gain. Patients with severe mental illness are at increased likelihood of developing weight-related comorbidities, particularly type II diabetes mellitus.

Many patients with severe and enduring mental illness do not regularly access primary care services. Admission to the psychiatric ward therefore provides an opportunity to address, not only the patient's mental health issues, but also any physical health issues.

**Method:**

A list of all inpatients on the eight general adult wards was obtained on 3rd of December 2020. Inpatients on the Psychiatric Intensive Care Unit were also incorporated, providing a final sample of 148 inpatients.

An audit tool was designed, to collect demographic data for each inpatient – gender, age, ethnicity, psychiatric diagnosis, as well as BMI on admission and, if applicable, whether a referral to the dietetics service was made.

**Result:**

Of the 148 inpatients, 91 were male, 57 female. Patient age ranged from 19 to 71 years. The majority were of “white British” ethnicity. The most common mental disorder diagnosis was schizophrenia (35 patients). For 14 of the 148 inpatients, no BMI was measured on admission. In the 134 inpatients that had BMI measured, 74 were in one of the “overweight”, “obese”, “very obese” and “morbidly obese” categories. Thirty-four patients met the criteria for requiring referral to the dietetics service. Of these, four were not referred, five were offered referral but declined, 17 referrals were made for other reasons, e.g. BMI <18 kg/m2, and one patient was referred despite no BMI being recorded.

**Conclusion:**

Current practice across the general adult inpatient wards in the trust indicates a proportion of patients do not have BMI recorded following admission. This may result in a valuable opportunity to address obesity being lost. There is a need to emphasise to ward staff the importance of recording BMI as part of the admission physical health screen and of the criteria for referring an inpatient to the dietetics service.

